# Integration of Genomic Tests in Prostate Cancer Care: Implications for Clinical Practice and Patient Outcomes

**DOI:** 10.3390/cimb46120864

**Published:** 2024-12-20

**Authors:** Christos Roidos, Anastasios Anastasiadis, Stavros Tsiakaras, Charalampos Loutradis, Panagiotis Baniotis, Dimitrios Memmos, Georgios Dimitriadis, Maria Papaioannou

**Affiliations:** 1 First Department of Urology, School of Medicine, Faculty of Health Sciences, Aristotle University of Thessaloniki, 541 24 Thessaloniki, Greece; drchriroid22@gmail.com (C.R.); aanastaa@auth.gr (A.A.); drstavros90@gmail.com (S.T.); dr.loutradis@gmail.com (C.L.); drbaniotis.p@gmail.com (P.B.); memmosdim@gmail.com (D.M.); 2 Laboratory of Biological Chemistry, School of Medicine, Faculty of Health Sciences, Aristotle University of Thessaloniki, 541 24 Thessaloniki, Greece

**Keywords:** prostate cancer, biomarkers, prognosis, risk stratification, mortality, Prolaris, Promark, OncotypeDx, Decipher

## Abstract

Prostate cancer (PCa) is a common malignancy in men and is among the leading causes of cancer-related death worldwide. Genomic tests assess disease aggressiveness and guide treatment, particularly in low- and intermediate-risk PCa. We reviewed the literature on the use of four genomic tests (Prolaris^®^, Promark^®^, Oncotype DX^®^, and Decipher^®^) in assessing the prognosis of PCa and their use in treatment decision-making. Most of the studies showed that Prolaris^®^ has a strong correlation with biochemical recurrence, metastasis risk, PCa-specific mortality (PCSM), and pathological features. Similarly, three studies on Promark^®^ indicated a connection between results and pathological features in the subsequent prostatectomy, time to metastasis, and biochemical recurrence. Fourteen studies on Oncotype DX^®^ showed a clear correlation between high scores, death, and PCSM. One study found that routine biopsy pathology reports, combined with serum PSA levels, provide a risk assessment comparable to Oncotype DX^®^ testing. Results from 22 studies on Decipher^®^ were controversial. The test was associated with conservative management, suggesting that patients with a high GC score are more likely to need radiation after surgery. Comparative studies indicated that Oncotype DX^®^ is preferable for assessing PCSM, Decipher^®^ for predicting metastasis, and Prolaris^®^ for predicting recurrence. With the incidence rate of PCa dramatically increasing, genomic tests appear to be useful adjunctive precision medicine tools with significant potential in improving prognostic discrimination, facilitating better risk stratification, and guiding personalized treatment, especially in the intermediate-risk patient group. Large-scale, prospective, multi-sectional studies are required to validate the utility of these tests prior to their integration into clinical practice.

## 1. Introduction

Prostate cancer (PCa) is the fourth most common malignancy worldwide and ranks eighth in cancer-related mortality [1]. Since the advent of Prostate-Specific Antigen (PSA) screening, the PCa detection rate has increased dramatically, with the majority of the cases being low-risk cases, with better outcomes and minimal side effects when prompt, appropriate treatment is applied [2,3].

Biomarkers refer to measurable biological molecules that can serve as indicators of various physiological or pathological processes within an organism. These molecules encompass a wide range of substances, including specific cell types, proteins, metabolites, RNA, DNA mutations, polymorphisms, or epigenetic modifications. Biomarkers are used in biomedical research and in clinical practice to facilitate the diagnosis and monitor health status, as well as prognostic and predictive tools of response to medical interventions [4]. Biomarkers are critical in PCa, where they play a pivotal role in early detection, risk stratification, and treatment decision-making. To date, the following genomic tests are commercially available:

Prolaris^®^ is the commercial name of the cell cycle progression genomic test. It includes 46 genes associated with the control of cell division and proliferation, producing the Cell Cycle Progression (CCP) score, ranging from 0 to 10, that is proposed to be used for risk stratification in men with low or favorable intermediate-risk disease. The tissue examined by this test derives either from a prostate biopsy or from a Radical Prostatectomy (RP) specimen [5].

ProMark^®^ is a biopsy-based Prostate Cancer prognostic assay that utilizes a multiplex immunofluorescence imaging platform to quantify the values of eight protein biomarkers demonstrated to be relevant to PCa aggressiveness in men with Gleason 3 + 3 and 3 + 4 PCa. The biomarker values are incorporated into a risk score (ProMark Score; range: 1–100), indicating the likelihood of having a high-risk disease [6].

The Oncotype DX^®^ Genomic Prostate Score (GPS) test is based on the results of a quantitative reverse transcriptase polymerase chain reaction (RT-PCR) assay that measures the expression levels of 17 genes (12 cancer-related genes associated with cell organization, androgen signaling, proliferation, and stromal response, and 5 housekeeper genes). mRNA is extracted from microdissected tumor tissue obtained from fixed prostate core biopsies. This test is used for risk stratification in patients with low- and intermediate-risk PCa [7].

The Decipher^®^ test is a 22-gene prognostic tool that examines prostate tissue—deriving either from RP or prostate biopsy—for the expression of genes related to cell proliferation, differentiation, androgen receptor signaling, and immune response. The genomic classifier (GC) score, ranging from 0 to 1, provides risk stratification for localized PCa patients. The Decipher^®^ Prostate Genomic Classifier has been awarded a Level 2A evidence rating in the NCCN Clinical Practice Guidelines, the highest among molecular tests [8].

Although previous studies have explored individual genomic tests in PCa, offering valuable insights, there is no study so far to collectively review data on these four tests, indicating the need for an up-to-date and comprehensive perspective on their clinical potential.

The aim of this review is to evaluate the literature regarding the available genetic tests and assess their prognostic value as well as their possible clinical use in recognition of intermediate-risk PCa patients.

## 2. Materials and Methods

The PubMed and Cochrane Library were searched for the purpose of this review using the following keywords “Prolaris”, “OncotypeDx”, “Decipher”, “Promark”, “prostate cancer”, “risk”, “clinical decision”, “management” and “mortality”. These databases were last searched on 25/10/2024. This review is registered at the Open Science Framework (OSF) registry (registration https://doi.org/10.17605/OSF.IO/8PSQ4, accessed on 16 November 2024).

Following deduplication, two reviewers screened the abstracts and titles of identified records for eligibility. The full texts were retrieved and screened, and the study characteristics were extracted in a standardized form. Study characteristics, participant features, type of biopsy specimen, reported primary and secondary outcomes of each study, and study inclusion and exclusion criteria were extracted from each study.

Inclusion criteria consisted of randomized or non-randomized comparative trials, single-arm prospective or retrospective studies published in English, with objectively measured outcomes and a minimum follow-up of 2 years. Studies utilizing patient-reported outcomes or published in languages other than English were excluded.

### Data Collection and Risk of Bias Assessment

Data from the studies included in this review were extracted by C.R., D.M. and P.B. using a pre-defined data extraction form. To ensure reliability and completeness, the extracted data were subsequently verified by an additional member of the review team. In cases where multiple articles were derived from the same study population, only the most recent report was included.

A narrative format was used for evidence synthesis. Due to the quality and heterogeneity of the included studies, a quantitative synthesis of the evidence was not performed. Owing to variations in targets and methodological approaches among the included studies, statistical pooling and meta-analysis of the results were considered inappropriate.

## 3. Results

**Figure 1:**
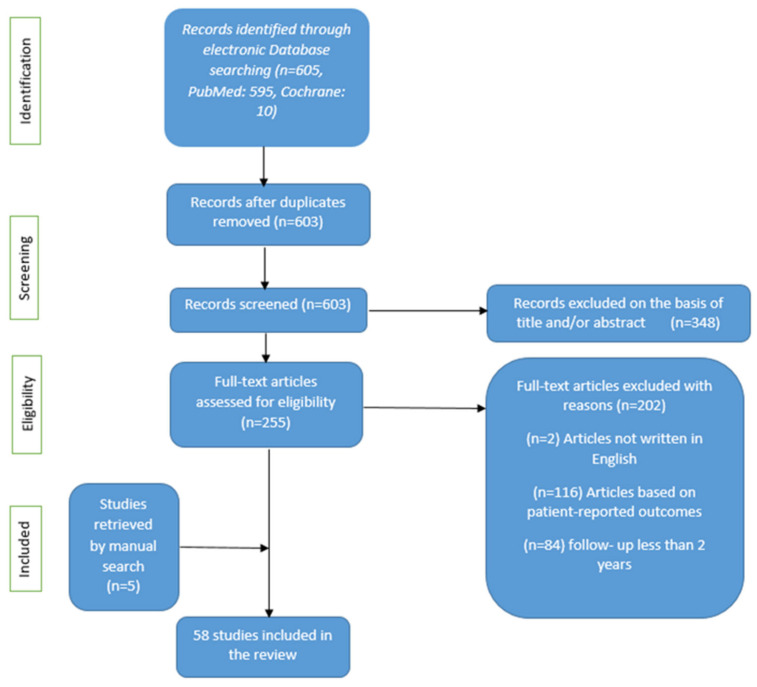
Flow diagram of the literature search.

A total of 605 records were identified through a systematic search of PubMed and the Cochrane Library. After deduplication, the search identified 603 studies from both the search engines used and 53 studies were included for further analysis after applying the aforementioned exclusion criteria (Figure 1).

These 53 studies were carefully reviewed and analyzed, with the key findings summarized in the tables and sections below, offering a comprehensive overview of the data relevant to the research objectives.

- Prolaris^®^

A total of 11 studies on Prolaris^®^ were included. A total of 4231 patients were included in these studies [9,10,11,12,13,14,15,16,17,18,19] (Table 1).

In six studies, the test was applied to specimens from prostate biopsies [9,13,16,17,18,19]; in five studies, tissue from RP [10,11,12,14,15], while in one of them, tissue from transurethral prostatectomy (TURP) was also used [10]. Three studies [16,17,18] were retrospective (27.2%), while the remaining eight [9,10,11,12,13,14,15,19] (72.8%) were prospective cohorts. The primary endpoint was biochemical recurrence (BCR) in six studies [11,12,13,14,15,18], risk of metastasis in two studies [11,19], and time-to-death in two studies [9,10]. Two studies examined the correlation of adverse pathologic (AP) features in the subsequent RP specimen and the CCP score in the previous biopsy [16,17]. Among the secondary endpoints were death and prostate cancer-specific mortality (PCSM) [10,13].

Multiple studies have demonstrated a statistically significant correlation between elevated CCP scores and poorer patient outcomes. Specifically, prostate cancer patients with higher CCP scores exhibit an increased risk of BCR, progression to metastatic disease, and PCSM [9,10,11,12,13,14,15,17,18,19]. Furthermore, elevated CCP scores following prostate biopsy have been associated with adverse pathological features [16,17], as well as a higher likelihood of extracapsular extension (ECE) during subsequent RP [16].

These findings support the utility of the Prolaris^®^ test in both risk stratification and prognostic assessment of PCa patients, aiding in therapeutic decision-making. However, the absence of double-blind, randomized controlled trials (DBRCTs) and studies evaluating the clinical application of this test remains a significant limitation.

- Promark^®^

Three studies with Promark^®^ were included in this review [6,20,21] (Table 2). A total of 857 patients were included in these studies. The study of Blume-Jensen et al. [6] was a non-interventional retrospective one. Specimens from prostate biopsies were used in this study, and its endpoints were the correlation between favorable pathology features at the time of RP and pre-operative Promark^®^ score. A lower test score was associated with favorable pathology in the subsequent RP specimen.

One prospective cohort was included. The test was applied to tissue derived from RP. The primary and secondary endpoints of this study were BCR and time to metastasis, respectively. Improved outcomes were observed, especially when the Promark^®^ test was used in combination with the NCCN Guidelines [20]. Finally, there was also a validation study, examining the match between the bioptic results of 293 early clinical practice patients and the previously conducted validation study, showing a match (confidence interval (CI): 95%).

The number of studies evaluating this test is limited, highlighting the need for further research to assess its clinical utility.

- Oncotype DX^®^

Fourteen studies and a total of 9146 patients referring to the Oncotype DX^®^ test were included in this review [22,23,24,25,26,27,28,29,30,31,32,33,34,35] (Table 3). Seven (50%) of them were prospective cohort studies [22,26,27,28,29,34,35]. The remaining 50% referred to retrospective observational studies. Only one study [25] used specimens from RP, while in the rest of the studies, the specimens used were derived from needle prostatic biopsies. BCR was the primary endpoint in two studies [22,27], time to BCR in two studies [32,33], while it was also the secondary endpoint in another study [28]. The risk of metastasis was examined in two studies [25,33]—one of them examining this in a 20-year horizon [25]. Time to metastasis was examined in one study as a primary endpoint [23] and in the other two studies as a secondary endpoint [22,32]. PCSM was examined in a study as a primary endpoint [25]. Time to PCa death was evaluated as the primary endpoint in one study [33] and as a secondary endpoint in two other studies [23,32]. These studies observed a statistically significant correlation between GPS score and BCR, time to BCR, risk of progression to metastatic disease, time to metastasis, time to PCa death, and PCSM [22,23,25,27,32,33]. These findings indicate that Oncotype DX^®^ can aid in accurate risk stratification and provides important prognostic information that can guide clinical decision-making.

Eight studies examined the association between higher scores of the genomic test applied in biopsy tissue and AP features in the subsequent RP specimen [24,26,28,29,30,31,34,35]. While the majority of them showcased a statistically significant correlation between GPS score and AP features at the subsequent RP, one of them [29] failed to prove this correlation. The same study examined the association of Oncotype DX^®^ with an upgrade in the surveillance biopsy results in patients under active surveillance (AS), but no statistically significant results were observed between the GPS score and the Gleason Score (GS) of the surveillance biopsy tissue. The study carried out by Greenland et al. [31] showed a correlation between the GPS score and the finding of the expansile cribriform subtype of prostatic adenocarcinoma. The study carried out by Murphy et al. examined the distribution of GPS in African American (AA) and European American (EA) men with localized PCa and showcased that the GPS independently predicts AP at RP in both AA and EA men, demonstrating comparable predictive accuracy and distribution across these populations [34].

The study conducted by Renavikar et al. found that risk stratification provided by information in the pathology report on routine biopsy assessment coupled with the serum PSA level, which is the current standard of care approach [8], is equivalent to that obtained by Oncotype DX^®^ testing [30]. The findings of this study question the importance of the implementation of Oncotype DX^®^ in PCa care.

- Decipher^®^

Twenty-two studies on the Decipher^®^ test were included in this review [36,37,38,39,40,41,42,43,44,45,46,47,48,49,50,51,52,53,54,55,56,57] (Table 4). These studies included a total of 15,206 patients. In 15 of them (68.2%), they examined specimens derived from RP [36,37,38,39,43,44,45,46,47,48,50,52,53,54,57]. In seven studies (31.8%), the genomic test was applied to tissue specimens from needle biopsy [40,42,49,51,55,56]. Twelve studies examined the chance of metastasis as their primary endpoint [36,37,38,39,40,44,45,46,47,52,53,55]. The findings of these studies show that there is a statistically significant correlation between the GC score and the probability of metastasis.

Nguyen et al. evaluated time to metastasis as the primary endpoint, with the cumulative incidence of metastasis over a 5-year interval serving as the secondary endpoint of their study [51]. The same secondary endpoint was examined in the study carried out by Freedland et al., showing statistically significant results [45].

AP features were the primary endpoint examined in four studies (18.2%) [41,42,48,54]. In two studies, a high GC score in bioptic tissue was associated with worse AP features in the subsequent RP [41,42]. In the other two studies, tissue from RP was used, and a high GC score was associated with worse AP features and disease prognosis [48,54].

PCSM was the primary endpoint in two studies [43,50] and the secondary endpoint in the other four studies [40,46,47,55], while death was the secondary endpoint in a study carried out by Klein et al. [38]. Overall survival (OS) was examined as the primary endpoint in one study [54] and as the secondary endpoint in the other two studies [46,55]. The findings of these studies showcase a statistically significant correlation between GC score and PCa prognosis, suggesting Decipher^®^’s use as a prognostic tool that can guide clinical decision-making [38,40,43,46,47,50,54,55].

Time to BCR was the endpoint in the study of White et al. The results were insignificant after logistic regression. Receiving salvage treatment, which served as the secondary endpoint of this study, was also not statistically significantly correlated to GC score after logistic regression [57].

A retrospective study conducted by Zaorsky et al. showed that the use of the Decipher^®^ test was associated with the selection of conservative management (Odds Ratio [OR] = 2.21, 95% CI = 2.04 to 2.38, *p* < 0.001). Among men who had high-risk GC scores and then had surgery, there was a three-fold higher chance of having worrisome findings in surgical specimens (OR = 2.94, 95% CI = 1.38 to 6.27, *p* = 0.005). The use of radiation after RP was statistically significantly associated with higher GC risk groups (OR = 2.69, 95% CI = 1.89 to 3.84) [49]. Nguyen et al. validated the results of three randomized controlled trials in an analysis, being the first validation of a gene expression biomarker on pretreatment prostate cancer biopsy samples from prospective randomized trials and demonstrating an independent association of GC score with distant metastasis (DM), PCSM, and OS [55]. A study by Ross et al. showed higher GC score patients benefited from adjuvant radiotherapy post-RP, in comparison with those with lower scores [53]. The findings of this study suggest that Decipher^®^ can play a crucial role in guiding clinical decision-making in the post-RP setting for PCa patients with AP features, such as pT3 disease or positive margins [53].

- Comparative studies

Three comparative studies were included in this review in total [58,59,60].

In a comparative study, including Prolaris^®^, Oncotype DX^®^, and Decipher^®^, Oncotype DX^®^ had poorer prognostic ability as far as metastasis prediction is concerned (Area under Curve (AUC): 0.65 vs. 0.73–0.74), with a significantly higher number need to diagnose (NND) (4.79 vs. 2.83–3.12). However, when it comes to PCSM prediction, Prolaris^®^’s performance suffered, while OncotypeDX^®^ showcased the lowest NND. When compared to clinical parameters risk stratification, all the above tests exceeded the competition’s performance [58].

Another study demonstrated a significant correlation between Decipher^®^ and Prolaris^®^ derived scores and between the 10-year probability of BCR reported by Prolaris^®^ and the 5-year probability of metastasis reported by Decipher^®^, which were highly correlated with each other. In a comparative retrospective cohort, both Prolaris^®^ and Decipher^®^ were significantly associated with metastasis outcome, but on multivariable analysis, only Decipher^®^ was a predictor of metastasis (Hazard Ratio (HR) 1.3, 95% CI [1.12–1.52], *p* = 0.0005) [59].

A genomic analysis of 231 biopsy cores from two ongoing prospective trials highlighted the heterogeneity within the tumor itself, implying a correlation between higher genomic scores and higher GS/tumor volume. Although its primary endpoint is related to the optimization of tumor selection for biopsy, statistical analysis of the genomic scores derived from the three aforementioned tests showed no statistically significant difference between them [60].

## 4. Discussion

In the context of deciding between treatment and AS for biopsy-positive PCa patients, several genomic tests are available to assist in risk assessment [8]. These include Oncotype DX^®^, Prolaris^®^, ProMark^®^, and Decipher^®^. Each of these tests provides valuable insights into the aggressiveness of the disease, helping clinicians and patients make informed decisions about the most appropriate management strategy.

These tissue-based prognostic biomarkers, evaluated in diagnostic biopsies, have shown correlations with AP findings in RP specimens, time to BCR, DM, and PCSM. Their potential to inform decisions between AS and active treatment is promising.

In this context, previous studies have examined the role of these genomic tests in guiding treatment decisions. Crawford et al. found that lower CCP scores from Prolaris^®^ were associated with a less aggressive therapeutic approach, though this study was based on patient questionnaires, limiting the robustness of its conclusions [61]. Similarly, Shore et al. reported findings consistent with those of Crawford et al., though the use of questionnaires introduces potential biases [62].

In a retrospective analysis using a large patient database, Decipher^®^ Biopsy scores were found to influence time spent on AS. Patients with high-risk GC scores remained on surveillance for a median of 13.6 months, significantly less than those with low/intermediate risk scores (median 33.0 months; *p* < 0.001). High-risk GC scores were also associated with a shorter time to treatment failure (TTF; *p* = 0.007) [63].

In a recent prospective study, Yazid Belkacemi and co-authors investigated how the Oncotype DX^®^ GPS influenced therapeutic decision-making for intermediate-risk PCa patients. Combining GPS with NCCN clinical risk factors, rather than relying solely on NCCN clinical risk stratification, resulted in notable reclassification, with 60% of patients moving to a higher-risk category. This likely would have resulted in substantial differences in treatment approaches [7].

These tests, mostly Decipher^®^ and secondarily Prolaris^®^, have also been tested in the post-RP setting. Their findings propose a correlation between the tests’ scores and the disease progression and conclusion. Only a few studies have examined the utilization of these tests in the treatment selection post-RP.

For instance, an additional analysis of the NRG/RTOG 9601 Randomized Clinical Trial aimed to clinically validate the GC of Decipher^®^ in men with recurrent PCa after RP. The GC, analyzed as a continuous variable (per 0.1 unit), demonstrated significant association with the primary endpoint, DM (HR: 1.26; 95% CI: 1.12–1.41; *p* < 0.001), and the secondary endpoints, PCSM (HR: 1.51; 95% CI: 1.30–1.77; *p* < 0.001), and OS (HR: 1.21; 95% CI: 1.10–1.33; *p* < 0.001). The clinical question of this study was whether the addition of short-term androgen deprivation therapy (ST-ADT) in high GC patients could be beneficial. The acquired data point to a positive answer [64].

Appropriate risk stratification is essential to avoid overtreatment in low-risk PCa patients while ensuring that high-risk patients receive necessary interventions. However, the challenge lies in balancing optimal treatment with the risk of undertreatment.

However, the hot patient group to whom the application of these tests should be beneficial and make a difference is the intermediate-risk group. In a clinical setting, it is of high importance to know whether radical treatment should be applied to these patients. Ideally, radical treatment should be offered only to those patients who are going to benefit from it. These biomarkers have been proposed as helpful tools for this aim. The aforementioned trials are not all focused on this group of patients, and the total quality of the evidence is not uncontested. Thus, in a blurry field, the question of whether the application of these biomarkers can overperform or add any prognostic value to the existing prognostic criteria remains.

The genomic tests reviewed in this article primarily focus on localized PCa. Current evidence does not address their clinical utility in metastatic or regionally node-positive cases. However, other genomic tests, such as AR-V7 testing, have shown potential relevance for metastatic PCa, particularly in predicting resistance to androgen receptor-targeted therapies [65]. Expanding research into the prognostic and predictive roles of these and other genomic tests in advanced stages of the disease is crucial for guiding personalized treatment strategies.

The wide adoption of these tests in clinical practice faces limitations, particularly concerning accessibility and cost. For instance, the Prolaris^®^ test offers prognostic value but is not universally accessible due to high costs and inconsistent insurance coverage, creating disparities in availability across healthcare systems [66]. Similarly, Blume-Jensen et al. emphasize the resource-intensive nature of assays, such as ProMark^®^, requiring specialized infrastructure and expertise, which limits their use in under-resourced settings [6]. Ebell further highlights that out-of-pocket costs for these tests can exceed USD 3000, presenting a significant financial burden for patients and healthcare systems [5]. Moreover, studies, including Belkacemi et al., highlight the variability in coverage policies and the lack of standardized guidelines for integrating these tests into clinical settings [7], as also noted in the NCCN guidelines [8].

Further high-quality studies are needed to determine whether these tests can consistently surpass or enhance the prognostic accuracy of existing criteria. Additionally, comprehensive evaluations of their cost-effectiveness and accessibility are imperative before they can be widely adopted in clinical practice.

In conclusion, while genomic tests such as Decipher^®^ and Prolaris^®^ show promising results in improving PCa management, especially in the intermediate-risk group, more large-scale, prospective trials are required. These tests have the potential to enhance existing prognostic criteria, but further validation is needed before they can be fully integrated into clinical practice.

The limitations of this review stem primarily from the heterogeneity of the studies included, which vary in design, sample size, and patient demographics, making direct comparisons challenging. Additionally, many of the studies rely on retrospective data or questionnaire-based methods, which may introduce biases and affect the generalizability of the findings. The relatively short follow-up periods in some studies limit the ability to assess long-term outcomes such as OS and PCSM. Furthermore, while genomic tests show promise in risk stratification, the clinical utility of these tools remains somewhat unclear due to limited evidence from large-scale, randomized controlled trials.

## 5. Conclusions

PCa is a highly prevalent malignancy, with the majority of cases classified as low-risk. Appropriate risk stratification is vital for avoiding over-treatment in low-risk patients and guiding treatment strategies in high-risk patients. Regarding the group of intermediate-risk patients, the choice of appropriate treatment can be more equivocal. The genomic tests reviewed serve as valuable adjunct tools in this regard, though the available studies remain inconclusive.

## Figures and Tables

**Table 1:** Studies referring to Prolaris^®^. HR: hazard ratio, meta: metastasis, RP: radical prostatectomy, BCR: biochemical recurrence, GS: Gleason Score, TURP: transurethral prostatectomy, PCSM: prostate cancer-specific mortality, pts.: patients ECE: extra-capsular extension, CCR: cell cycle risk, path.: pathology OR: odds ratio, AS: active surveillance, WW: watchful waiting, CCP: cell cycle progression score.

Trial	Type	Pts (N)	Specimen	Endpoint	Outcome	Sec. Endpoint	Sec. Outcome
Cuzick et al., 2015 [9]	Cohort	761	Biopsy	Death	HR: 2.17		
Cuzick et al., 2011 [10]	Two cohorts	410 + 429	RP + TURP	Time to BCR + death	HR: 1.74 + 2.92	Death	HR: 2.99
Bishoff et al., 2014 [11]	Three cohorts	582	RP	BCR + meta	HR: 1.47/4.19		
Tosoian et al., 2017 [12]	Cohort	236 (l)	RP	BCR	HR: 1.77		
Freedland et al., (2013) [13]	Cohort	141	Biopsy	BCR	HR: 2.55	PCSM	HR: 3.77
Oderda et al., 2017 [14]	Cohort	52	RP	BCR	HR: 5.54 (high-risk pts) 2.64 (low-risk pts)		
Cooperberg et al., 2013 [15]	Cohort	413	RP	BCR	HR: 2.1/1.7 (adj.)		
Wibmer et al., 2019 [16]	Retrosp. Analysis	118	Biopsy	Assoc. ECE to CCR	Worse path. feat		
Morris et al., 2021 [17]	Retrosp. Observ.	222	Biopsy	Post RP GS	OR: 4.10/3.72		
Shangguan et al., 2020 [18]	Retrosp. Analysis	100	Biopsy	BCR	HR: 1.373		
Canter et al., 2019 [19]	Cohort (AS vs. WW)	767	Biopsy	Meta	HR CCP: 2.04 HR CCR: 3.86		

**Table 2:** Studies referring to Promark^®^. HR: hazard ratio, BCR: biochemical recurrence, meta: metastasis, RP: radical prostatectomy, GS: Gleason Score, AUC: area under curve.

Trial	Type	Pts (N)	Specimen	Endpoint	Outcome	Sec. Endpoint	Outcome
Blume-Jensen et al., 2015 [6]	Non-interventional retrospective	276	Biopsy matched to RP	Favorable pathology (GS ≤ 3 + 4, T ≤ T2)	AUC 0.65 *p* < 0.0001	GS ≤ 3 + 3 T ≤ T3a	AUC 0.68 *p* < 0.0001
Saad et al., 2017 [20]	Cohort	288	RP	BCR	HR: 1.724 (improved + NCCN)	Time to meta	HR: 10.71
Choudhury et al., 2015 [21]	Validation study	293	Biopsy	Match to early clinical practice and validation study	Match CI: 95%		

**Table 3:** Studies referring to Oncotype DX^®^. BCR: biochemical recurrence, AP: adverse pathology, adj.: adjuvant, HR: hazard ratio, OR: odds ratio, meta: metastasis, RP: radical prostatectomy, PCD: prostate cancer death, EPE: extra-prostatic extension, SVI: seminal vesicle invasion, DM: distant metastasis, GS: Gleason Score, PCSM: prostate cancer-specific mortality, EBRT: external beam radiation therapy, AA: African American, EA: European American.

Trial	Type	Pts (N)	Specimen	Endpoint	Outcome	Sec. Endpoint	Outcome
Cullen et al., 2015 [22]	Cohort	431	Biopsy	BCR AP	HR: 2.9 HR (adj): 2.7 OR (AP): 3.3	Time to meta	HR: 3.8
Van Den Eden et al., 2018 [23]	Retrospective	279 (RP treated)	Biopsy	Time to meta	HR: 2.75	Time to PCD	HR: 3.23
Covas Moschovas et al., 2022 [24]	Retrospective	749	Biopsy	AP after robotic RP	OR (EPE): 1.8 OR (SVI): 2.1		
Brooks et al., 2021 [25]	Retrospective	428	RP	20 yr DM, PCSM	HR: 2.24 HR: 2.30		
Eggener et al., 2019 [26]	Prospective	114	Biopsy	AP	OR: 2.2 OR (adj.): 1.9 OR (adj. NCCN): 2		
Helfand et al., 2022 [27]	Prospective	141	Biopsy	BCR post RP	HR: 2.14		
Kornberg et al., 2019 [28]	Prospective	215	Biopsy	AP post delayed RP	HR: 1.16	BCR	HR: 1.1
Lin et al., 2020 [29]	Prospective	432	Biopsy	AP post delayed RP	Not statistically significant	Upgrade in surveillance biopsy	Not statistically significant
Renavikar et al., 2023 [30]	Retrospective-comparative	4967 (181 tested)	Biopsy	Concordance clinicopathologic vs. Oncotype DX	high		
Greenland et al., 2019 [31]	Retrospective	296 (319 biopsies)	Biopsy	AP	Expansile cribriform		
Canter et al., 2023 [32]	Retrospective	450 (RT)	Biopsy	Time to BCR	HR: 3.08	Time to DM, PCa death	HR: 5.19 HR: 13.07
Janes et al., 2023 [33]	Retrospective	238 (EBRT)	Biopsy	Time to BCR, DM, PCD	HR: 3.62 HR: 4.48 HR: 5.36		
Murphy et al., 2020 [34]	Prospective	172	Biopsy	AP (adj. race, NCCN)	OR (AA): 4.58 OR (EA): 4.88		
Salmasi et al., 2018 [35]	Prospective	134	Biopsy	AP (RP in 6 months)	OR: 3.28 wide and overlapping distribution among PIRADS scores		

**Table 4:** Studies referring to Decipher^®^. RP: radical prostatectomy, meta: metastasis, AUC: area under curve, OR: odds ratio, ART: adjuvant radiotherapy, SRT: salvation radiotherapy, GC: genomic classifier score, HR: hazard ratio, BCR: biochemical recurrence, RT: radiation therapy, ADT: androgen deprivation therapy, PCSM: prostate cancer-specific mortality, AP: adverse pathology, NPV: negative predictive value, CAPRA: cancer of the prostate risk assessment score, F-IR: favorable intermediate risk, VL/LR: very low/low risk, PSA: prostate-specific antigen, LNI: lymph node involvement, GS: Gleason Score, OS; overall survival, DM: distant metastasis.

Trial	Type	Pts (N)	Specimen	Endpoint	Outcome	Sec. Endpoint	Outcome
Karnes et al., 2013 [36]	Case-cohort	256	RP	Meta	AUC: 0.75		
Den et al., 2014 [37]	Cohort	188	RP	Meta post ART/SRT	If GC > 0.4, ART: 6% SRT: 23% (*p* < 0.01)		
Klein et al., 2016 [38]	Cohort	57	RP	Meta	8 (14%) HR: 1.72	Death	3 (5.3%)
Ross et al., 2014 [39]	Cohort	85	RP	Meta after BCR	AUC 0.82 *p* = 0.003		
Nguyen et al., 2017 [40]	Cohort	235 (105 RP, 130 RT ± ADT)	Biopsy	Meta	HR: 1.37	PCSM	HR: 1.57 5 yr: 0–94%
Kim et al., 2019 [41]	Retrospective	266	Biopsy	AP	OR: 1.29 NPV: 91% + CAPRA: AUC: 0.65		
Herlemann et al., 2020 [42]	Retrospective	647	Biopsy	AP at RP	OR: 1.34 per 0.1. F-IR with low score no worse than VL/LR		
Karnes et al., 2018 [43]	Cohort	561	RP	PCSM in 10 years	OR: 3.91 3.96 high 3.06 BCR2 1.95 MET		
Spratt et al., 2018 (ii) [44]	Cohort	477	RP	Meta	HR: 5.95 4.26 (PSA) 12.2 (LNI)		
Freedland et al., 2016 [45]	Retrospective	170	RP	Meta (SRT post RP)	HR: 1.58 per 0.1 c-index: 0.85	5 yr cumulative	L: 2.7% M: 8.4% H: 33.1% (*p* < 0.001)
Feng et al., 2021 [46]	Randomized cohort (phase 3 trial)	352	RP	DM	HR:1.17	PCSM OS	HR: 1.39 HR: 1.17
Ross et al., 2016 [47]	Retrospective case-cohort	356	RP	Meta	HR: 1.26	PCSM	*p* < 0.01
Klein et al., 2017 [48]	Retrospective	337	RP (GS: 3 + 3)	AP	13% med 7% high pT2: 0.23 pT3: 0.3		
Zaorsky et al., 2023 [49]	Retrospective	8927	Biopsy	Conservative management	OR: 2.21	Local therapy (LR, IF-R) AP after RP RT after RP	OR: 4.79 OR: 2.94 OR: 2.69
Cooperberg et al., 2015 [50]	Retrospective	185	RP	PCSM (CAPRA-S vs. GC)	HR: 1.36 2.36 (high) HR: 1.86 11.26 (high)		
Nguyen et al., 2017 [51]	Retrospective	100 (55 IR, 45 high)	Biopsy	Time to DM	HR: 1.4 per 0.1	Cumulative incidence 5 yr meta	20%, if GC > 0.6
Klein et al., 2015 [52]	Retrospective	169 (AP post-RP)	RP	DM	OR: 1.48 c-index: 0.77		
Ross et al. (2016) (ii) [53]	Retrospective comparative	422	RP	Meta (ART vs. SRT vs. NO)	Low GC low rate of meta. High GC benefit from ART		
Treacy et al., 2023 [54]	Retrospective	695	RP	AP 0S PFS	Gene overexpression prognostic value (*p* < 0.05)		
Nguyen et al., 2022 [55]	3 randomized phase-3 trials	385	Biopsy	DM	HR: 1.22	PCSM OS	HR: 1.23 HR: 1.12
Press et al., 2022 [56]	Retrospective cohort	133 (AS)	Biopsy	Biopsy upgrade	OR: 1.37 per 0.1		
White et al., 2021 [57]	Observational	203	RP	Time to BCR	Not significant after logistic regression	Receipt of salvage treatment	Not significant after logistic regression
